# LIPOPHAGY: a novel form of steroidogenic activity within the LEYDIG cell during the reproductive cycle of turtle

**DOI:** 10.1186/s12958-019-0462-2

**Published:** 2019-02-09

**Authors:** Imran Tarique, Waseem Ali Vistro, Xuebing Bai, Ping Yang, Chen Hong, Yufei Huang, Abdul Haseeb, Enxue Liu, Noor Samad Gandahi, Mengdi Xu, Yifei Liu, Qiusheng Chen

**Affiliations:** 1 MOE Joint international Research Laboratory of Animal Health and Food safety, College of Veterinary Medicine, Nanjing Agricultural University, Nanjing, 210095 Jiangsu Province China; 2 Faculty of Veterinary and Animal Sciences, University of Poonch Rawalakot, Azad Kashmir, Pakistan

**Keywords:** Leydig cell, Lipophagy, Hibernation, LC3, 3β-HSD, Chinese soft-shelled turtle

## Abstract

**Background:**

Steroidogenesis is an indispensable process that is indirectly associated with spermatogenesis in the Leydig cell (LC) to utilize the lipid droplets (LDs) that are critical to maintaining normal testosterone synthesis. The regulation of LD mobilization, known as lipophagy, in the LC is still largely unknown.

**Method:**

In the present study, the LC of the Chinese soft-shelled turtle was investigated to identify the steroidogenic activity and lipophagy during the annual reproductive cycle by light microscopy, immunohistochemistry (IHC), immunofluorescence (IF), and transmission electron microscopy (TEM).

**Results:**

The LC showed a dynamic steroidogenic function with strong activity of 3β-HSD, vimentin and tubular ER during hibernation by IHC and TEM. The tubulo-vesicular ER had a weak immunopositive reaction for 3β-HSD in the LC during reproductive phase, suggesting persistent steroidogenic activity. ORO staining and TEM demonstrated that a larger number of LDs had accumulated in the LC during hibernation than in the reproductive phase. These LDs existed in close association with mitochondria and lysosomes by being dynamically surrounded by intermediate filaments to facilitate LD utilization. Lysosomes were found directly attached to large LDs, forming an autophagic tube and engulfing LDs, suggesting that micro-lipophagy occurs during hibernation. Furthermore, the IHC of ATG7 (Autophagy Related Gene 7) and the IF of the LC3 (Microtubule-associated protein light chain 3), p62 (Sequestosome-1 (SQSTM1) and LAMP1(Lysosomal-associated membrane protein 1) results demonstrated strong expression, and further confirmation by TEM showed the existence of an autophagosome and an autolysosome and their fusion during the hibernation season.

**Conclusion:**

In conclusion, the present study provides clear evidence of LD consumption in the LC by lipophagy, lysosome and mitochondria during the hibernation period, which is a key aspect of steroidogenesis in the Chinese soft-shelled turtle.

## Background

The adult male testis has two significant roles, the production of spermatozoa and the secretion of sexual steroids [1]. Like other vertebrates, these two important functions in reptiles occur in the tubular and interstitial compartments of the testis, respectively. Spermatogenesis occurs within the seminiferous tubules (ST). However, the male specific, Leydig cells (LC), which are distributed in the interstitial tissues are accountable for androgen hormone production and play a critical role in spermatogenesis [2]. It is well-known that many mammals have an associated reproductive pattern. In some seasonally breeding reptilian species, separate hormonal cues initiate spermatogenesis and mating, which is so-called dissociated reproductive pattern [3–5]. Spermatogenesis in the Chinese soft-shelled turtle is an annual event that is active in late spring, summer and fall (May to October) and ends with one massive release of sperm in late October or early November. This species remains quiescent (the hibernation period) throughout the rest of the year (December to April) [6]. During the testicular cycle, structural changes in the male germinal epithelium have been associated with fluctuating reproductive patterns and variation in LC activity [7–9]. Previously, our research group produced detailed cellular work on the germ cell, Sertoli cell and their novel role during the annual cycle [10, 11], but an ultrastructural analysis of the LC in the Chinese shelled-turtle has not been reported.

Leydig cells are characterized by lipid droplets and composed of neutral lipid cores, mainly made up of cholesteryl esters and triglycerides [12]. The sequestration of lipids in droplet form provides a depot of stored energy that can be accessed in a regulated fashion according to cellular need. Recently, it has been reported that autophagy regulates testosterone biosynthesis [13] by facilitating cholesterol uptake [14] and regulating fertility [15] in rats. Autophagy of lipid droplets, or lipophagy is an alternative to lipolysis. It is characterized by a spherical double membrane structure known as an autophagosome. The autophagosome is subsequently fused with the lysosome to form an autolysosome. The contents of the autolysosome are then degraded by the lysosomal hydrolytic enzymes, which release the contents into the cytoplasm [16]. Another type of autophagy is microlipophagy, which is characterized by the direct interaction of lysosome and the lipid droplet, where the contents enter the lysosome through an invagination or deformation of the lysosomal membrane [17]. During the past few decades, the interaction between lysosomes and lipid droplets have become a focus for research. The lysosome is a key organelle in autophagy and coordinates the sorting, recycling and delivery of endogenous lipids [18].

The LC3 and p62 biomarkers are widely used for monitoring autophagy [19]. LC3 is a soluble protein which is distributed ubiquitously in mammalian tissues. During autophagy, LC3-I is conjugated to phosphatidylethanolamine to form an LC3-phosphatidylethanolamine conjugate (LC3-II), which is tightly bound to the autophagosomal membranes [20]. The p62 protein, also called sequestosome 1 (SQSTM1), helps with the recognition of autophagic cargo in numerous cell types [21]. ATG 7 is one important member of an autophagy-related gene family that encodes the E1-like enzyme, which facilitates both LC3 and other autophagy- related genes [22–24]. The well-known proteins called LAMP1 and LAMP2 are commonly used for lysosomes. Recently, it has been suggested that LAMP1 binds to fatty molecules and cholesterol is involved in lysosomal cholesterol dissemination [25]. Together with these findings, Leydig cells and autophagy have also been studied in mammals by using the traditional method of electron microscopy [26, 27].

Autophagy is a conserved system among eukaryotes and is well-studied in mammals [12]. It also contributes to the adaptive response to starvation and various, extrinsic and intrinsic stresses. Nevertheless, its role in reptilian Leydig cells is still largely unknown. The Chinese soft-shelled turtle (*Pelodiscus sinensis*) is one of the most economical and pharmacologically worthy reptiles in China. As a seasonally breeding animal, this species is an excellent model for studying the regulation of reproductive activity. The objectives of the current study were to analyze the seasonal ultrastructural changes in the Leydig cell and to explore lipophagy and the consumption of LDs within the testis of the Chinese soft-shelled turtle.

## Methods

### Experiment animals

*Pelodiscus sinensis* soft-shelled turtles (mature males, > 3 years of age) were purchased from an aquatic farm in the Nanjing, Jiangsu province of China in March and October, with six turtles for each time period. The animals were rendered comatose using intraperitoneally administered sodium pentobarbital (20 mg/animal) and were sacrificed by cervical dislocation. The testes were collected immediately and fixed to perform the different techniques (details below).

### Light microscopy

The testis samples were placed in 10% neutral buffered formalin for fixation overnight, and then embedded in paraffin wax. Sectioning was done at 5 μm. These sections were stained with haematoxylin and eosin procedures (Harry’s haematoxyline for 2 min and 1% eosin for 30 s). For light microscope analysis using an Olympus microscope (BX53) and camera (Olympus DP73, Japan).

### Oil red O (ORO) staining

Testis samples (5 μm thick frozen slices) were washed with PBS, fixed with 4% formaldehyde for 10 min, and stained with ORO staining (Sigma) solution (oil O saturated solution in isopropanol: water, 3:2) for 15 min. After staining slides, washed with warm distilled water (37 °C) for 15 min. Subsequently, counterstained with hematoxylin for 2 min, and were rinsed with tap water for 60 s.

### Immunohistochemistry (IHC)

The paraffin sections prepared on glass slides were briefly deparaffinized and washed with phosphate buffered saline (PBS). To block any further activity forms of endogenous peroxidases, the sections were treated with 3% hydrogen peroxide (H_2_O_2_) in PBS for 15 min at 37 °C. The samples were then treated with 5% bovine serum albumin (BSA 5%) and incubated with a primary antibody (Table 1) in a moisture chamber at 4 °C for 24 h, while PBS (pH 7.2) served as the negative control. After washing, the sections were incubated with the secondary antibody for 1 h at room temperature. The sections were then rehydrated in PBS (pH 7.2) and incubated with an avidin-biotinylated peroxidase complex for 45 min at 37 °C. After being washed with PBS, peroxidase activity was revealed using DAB (Boster Bio-Technology Co., LTD), according to the manufacturer’s instructions.

**Table 1:** The information for primary and secondary antibodies

	Species	Catalog No.	Dilution	Source
Primary antibodies				
Vimentin	Rabbit	Bs-0756R	1:100	Bioss ANTIBODIES
3Beta-HSD	Rabbit	A1823	1:100	ABclonol Technology
LAMP1	Rabbit	55,273–1-AP	1:100	Proteintech
ATG7	Rabbit	10,088–2-AP	1:100	Proteintech
LC3	Rabbit	12,135–1-AP	1:100	Proteintech
P62	Rabbit	51,145	1:100	Cell Signaling Technology
Secondary antibodies				
AlexaFlour 488 Gaot anti-rabbit IgG		FMS-RBaf48801	1:100	FCMACS
Anti-rabbit igG		KIT-5004/5/6		MXB Biotechnology

### Immunofluorescence (IF)

The paraffin sections were incubated with a primary antibody overnight at 4 °C. Following the primary antibody (Table 1) application, all the samples were incubated with a secondary antibody for 1 h at 4 °C and were rehydrated in PBS. For negative control PBS (pH 7.2) was used. The sections were incubated with DAPI (4′, 6-diamidino-2-phenylindole = nuclear staining) and were stimulated under a fluorescence microscope over time. All the specimens were initially viewed using an LED to visualize the fluorescence under the microscope. Photographs were taken of every section of the specimen at 40-400x magnification power.

### Transmission electron microscopy (TEM)

For the ultrastructural analysis, testis specimens were cut into small parts (1 mm^3^) and fixed in 2.5% (*v*/v) glutaraldehyde in 0.1 M phosphate-buffered saline (PBS;4 °C, pH 7.4; overnight). Thereafter, they were post-fixed with 1% (*w*/*v*) osmium tetroxide in the same buffer for 1 h and washed in the buffer. The samples were dehydrated with increasing concentrations of ethanol and infiltrated with a propylene oxide-araldite mixture for embedding into araldite. The blocks were sectioned. Ultrathin sections (50 nm) were mounted on Formvar-coated grids and stained with uranyl acetate and Reynolds led citrate for 20 min per step. The sections were analyzed with TEM (Hitachi H-7650; Japan).

### Statistical analysis

To performed histo-morphometry of hematoxylin and eosin stained sections, from each sample 5 μm serial sections were used. A total of 36 tissue sections with two replications of each analyses; equal numbers of tissue sections (*n* = 18) were taken from each group measured by Image Pro v10 and analyzed statistically and presented with Origin Pro 2018. The results were presented as the mean ± SEM. The statistical significance of differences among the means were analyzed by t-test (*P* < 0.05).

## Results

**Fig. 1:**
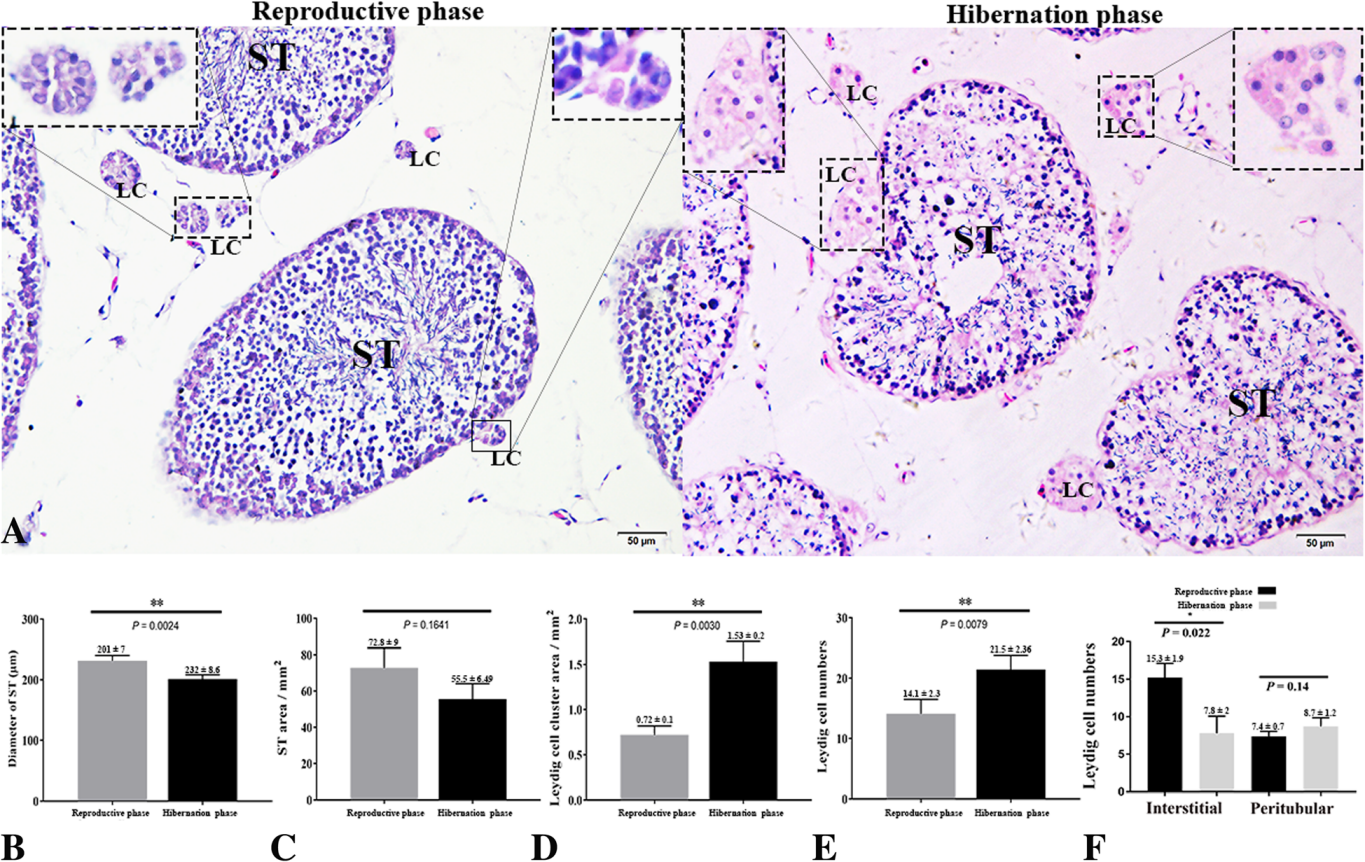
Testis histology during reproductive activity and hibernation. A higher magnification is illustrated in the rectangular area. Quantification of the (**b**), diameter of ST (*P* = 0.0024) (**c**), ST areas (*P* = 0.1641) (**d**), Leydig cell cluster area (*P* = 0.0030) (**e**), Leydig cell numbers (*P* = 0.0079) (**f**) and Leydig cell number at the peritubular (*P* = 0.14) and interstitium (*P* = 0.022) (*n* = 18 per group). ST: Seminiferous tubule; L: Leydig cell. Scale bar = 20 μm (**a**). Data presented as Mean ± SEM

Based on the histological analyses, the seminiferous tubules (ST) were lined with developing germ cells and permanent Sertoli cells during reproductive activity (Fig. 1a). Furthermore, a significantly higher diameter and area of ST were observed during the reproductive phase compared to the hibernation without a change in the ST area (Fig. 1b and c). During hibernation, the ST showed residual spermatozoa and cleared ad-luminal compartments with decreased sperm numbers (Fig. 1a). The Leydig cells in the testis of the Chinese soft-shelled turtle were characterized by a compact morphology during reproductive phase and a significantly increased area during hibernation (Fig. 1a and d). The numbers of LC were statistically higher during hibernation than in reproductive phase (Fig. 1e). Additionally, the LC numbers at the interstitial position were significantly higher than at the peritubular position during reproductive phase, while those, at peritubular position were relatively higher during hibernation (Fig. 1f).

**Fig. 2:**
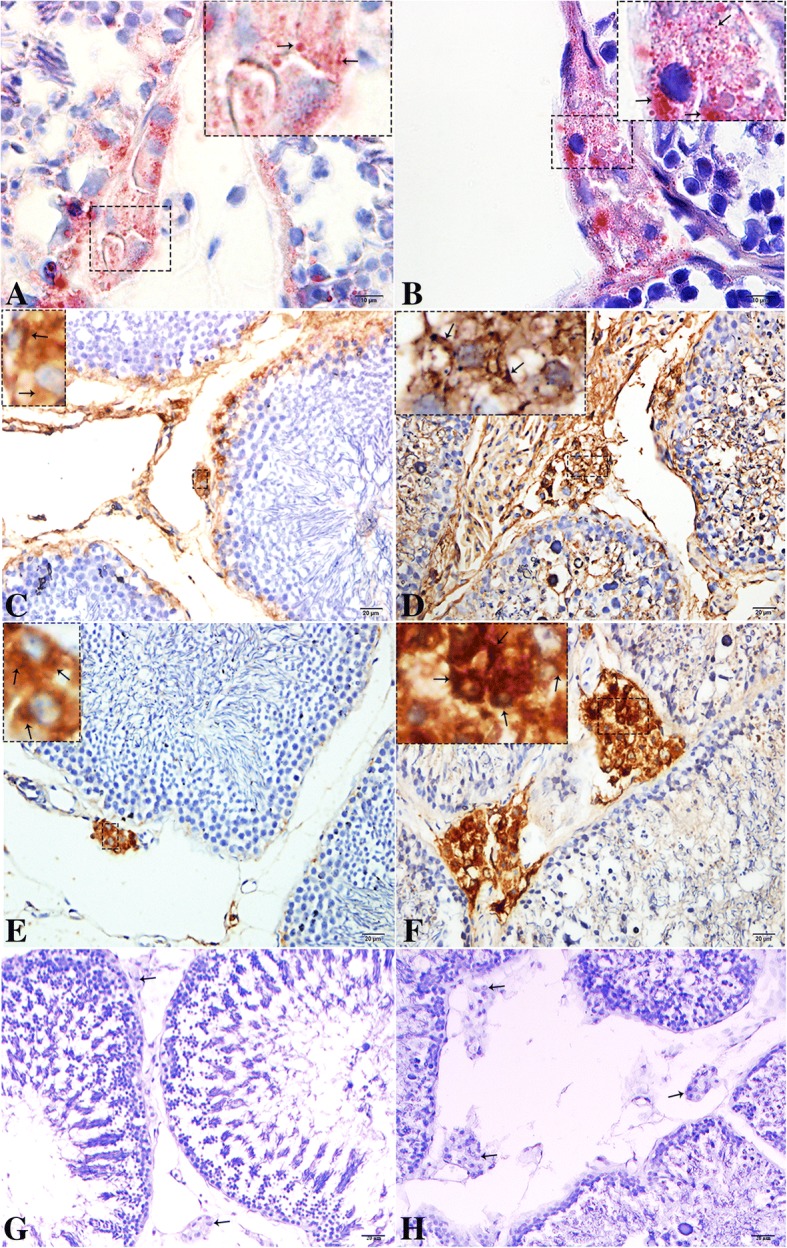
Light micrograph of Oil Red O staining and immunohistochemistry of vimentin, and 3β-HSD in the testis during resproductive and hibernation phase. (**a**-**b**) ORO staining of lipid droplets (arrow), (**c**-**d**) vimentin (**e**-**f**) 3β-HSD immunolocalization (arrow) and (G-H) negative control (arrow) in Leydig cell. A higher magnification is illustrated in the rectangular area. Scale bar = 10 μm (**a**-**b**) and 20 μm (**c**-**h**)s

The light microscopic results of the ORO staining indicate on small LD content within the LC during reproductive phase (Fig. 2a), while large amount of LD was observed during hibernation (Fig. 2b). Immunoreactivity of vimentin was also weak during reproductive phase compared to a strong immunosignal during hibernation. The immunopositive reaction mainly appeared at the cytoplasmic membrane and around the nucleus (Fig. 2b and c). 3Beta-HSD is a key enzyme in the biosynthesis of steroid hormone. Its weak to moderate labeling was observed during reproductive phase, while moderate to intense immunosignal occurred during hibernation. The immunolabeling mainly appeared at the cytoplasm and close to the nucleus (Fig. 2e and d).

**Fig. 3:**
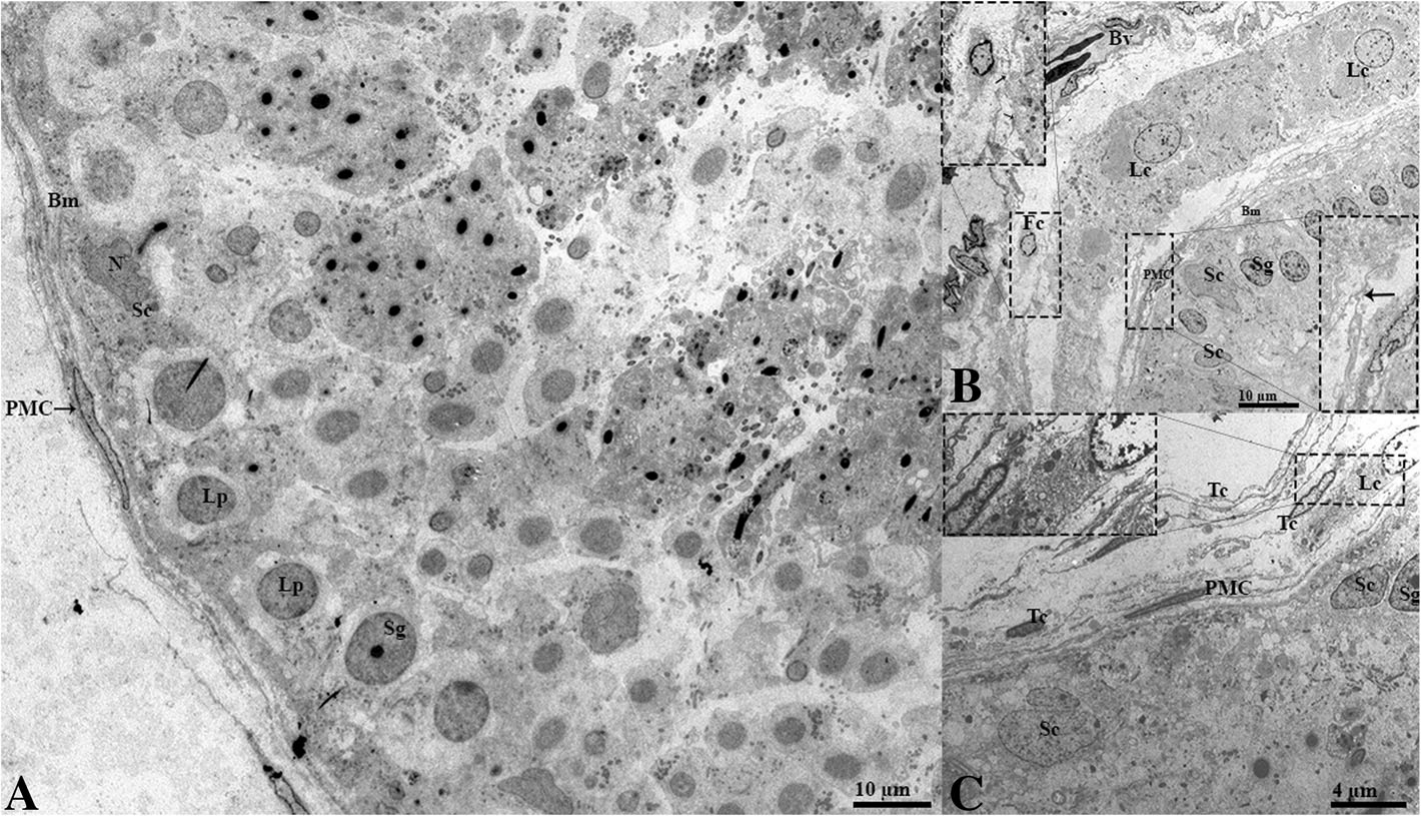
Electron micrograph of the seminiferous tubules and the interstitium of the testis during reproductive phase. (**a**) Seminiferous tubules containing the maturing spermatocytes in the ad-lumen, spermatogonia and Sertoli cell at the basement membrane surrounded by peritubular myoid cells. (**b**-**c**) Interstitium space consisting of the Leydig cell, telocytes, fibroblasts and blood vessels. The higher magnification illustrates the contact between different the cells. Bm: basement membrane; Sc: Sertoli cell; N: Nucleus; Sg: spermatogonia; PMC: Peritubular myoid cell; Lc: Leydig cell; N: nucleus; Fc: Fibroblast; Tc: Telocyte; Bv: Blood vessel. Scale bar = 10 μm (**a**-**b**) and 4 μm (**c**)

**Fig. 4:**
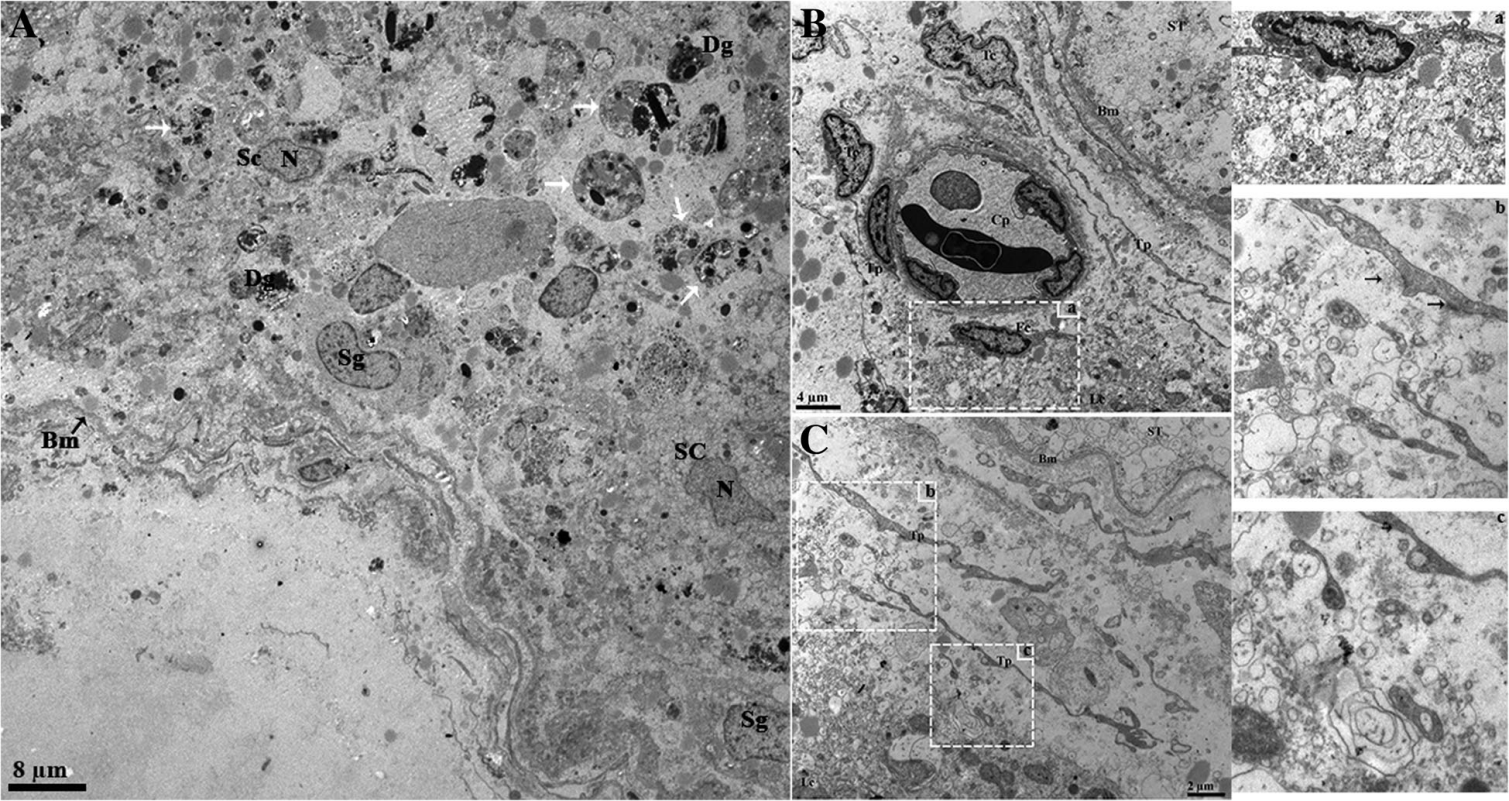
Electron micrograph of seminiferous tubules and the interstitium of the testis during hibernation. (**a**) Seminiferous tubules containing various residual spermatids and entotic vesicles (white arrow) in the ad-lumen, spermatogonia and Sertoli cell at the basement membrane. (**b**-**c**) Interstitium consists Leydig cell, Telocyte, fibroblast and capillary. The higher magnification illustrates the contact between the different cells (**a,b,c**). Bm: basement membrane; Sc: Sertoli cell; N: Nucleus; Sg: spermatogonia; PMC: Peritubular myoid cell; Lc: Leydig cell; N: nucleus; Fc: Fibroblast; Tc: Telocyte; Cp: Capillary. Scale bar = 8 μm (**a**), 4 μm (**b**), and 2 μm (**c**)

The ultrastructure of the seminiferous tubules during reproductive phase showed that the seminiferous epithelium was lined with numerous spermatids progressing into the various phases of spermiogenesis and that the lumen was filled with free spermatozoa (Fig. 3a). The interstitium showed the peritubular myoid cells, telocytes, and fibroblasts in contact with the LC. (Fig. 3b and c). In hibernation, the ST displayed depolarized residual spermatids, and the entotic vacuoles at the ad-luminal compartment were indicative of a clearing of previous spermatogenic residues (Fig. 4a). In the interstitial space, fibroblasts and telocytes displayed communication via vesicles with the LC (Fig. 4b and c).

**Fig. 5:**
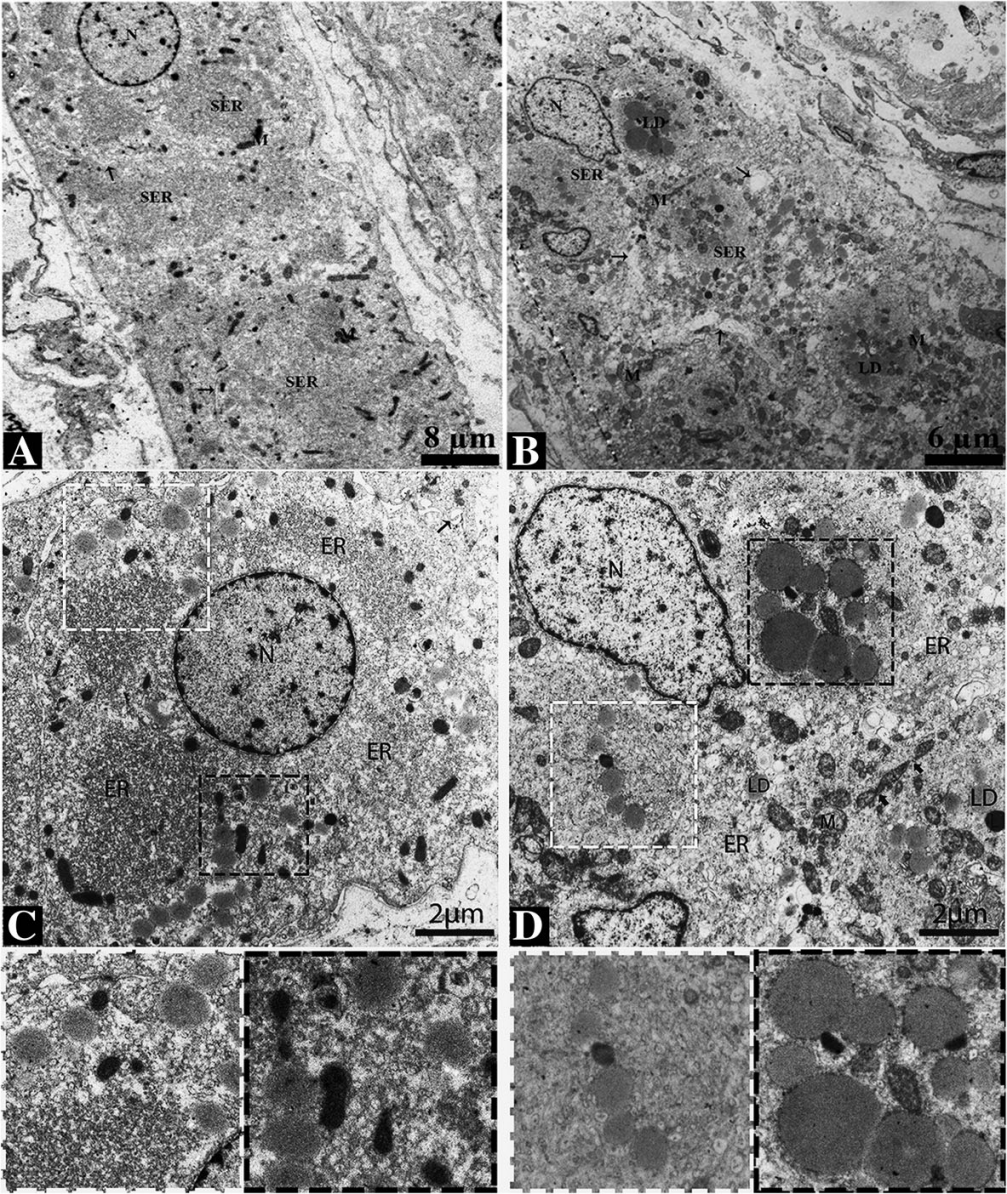
Electron micrograph of the Leydig cell in the testis of the Chinese soft-shelled turtle. The Leydig cell is compactly exhibited during reproductive phase (**a**) and loosely exhibited during hibernation phase (**b**) at the interstitium. The cytoplasm of the Leydig cell is rich in vesicular endoplasmic reticulum (**c**), lipid droplets, mitochondria and tubular endoplasmic reticulum (**d**). White thin arrow: gap junction; black thin arrow: finger-like prolongations in the interdigitation space; black thick arrow: dividing mitochondria. The illustration indicates a magnified view of the white and black square. N: nucleus; ER: endoplasmic reticulum; LD: lipid droplets; M: mitochondria. Scale bar = 8 μm (**a**), 6 μm (**b**) and 2 μm (**c-d**)

**Fig. 6:**
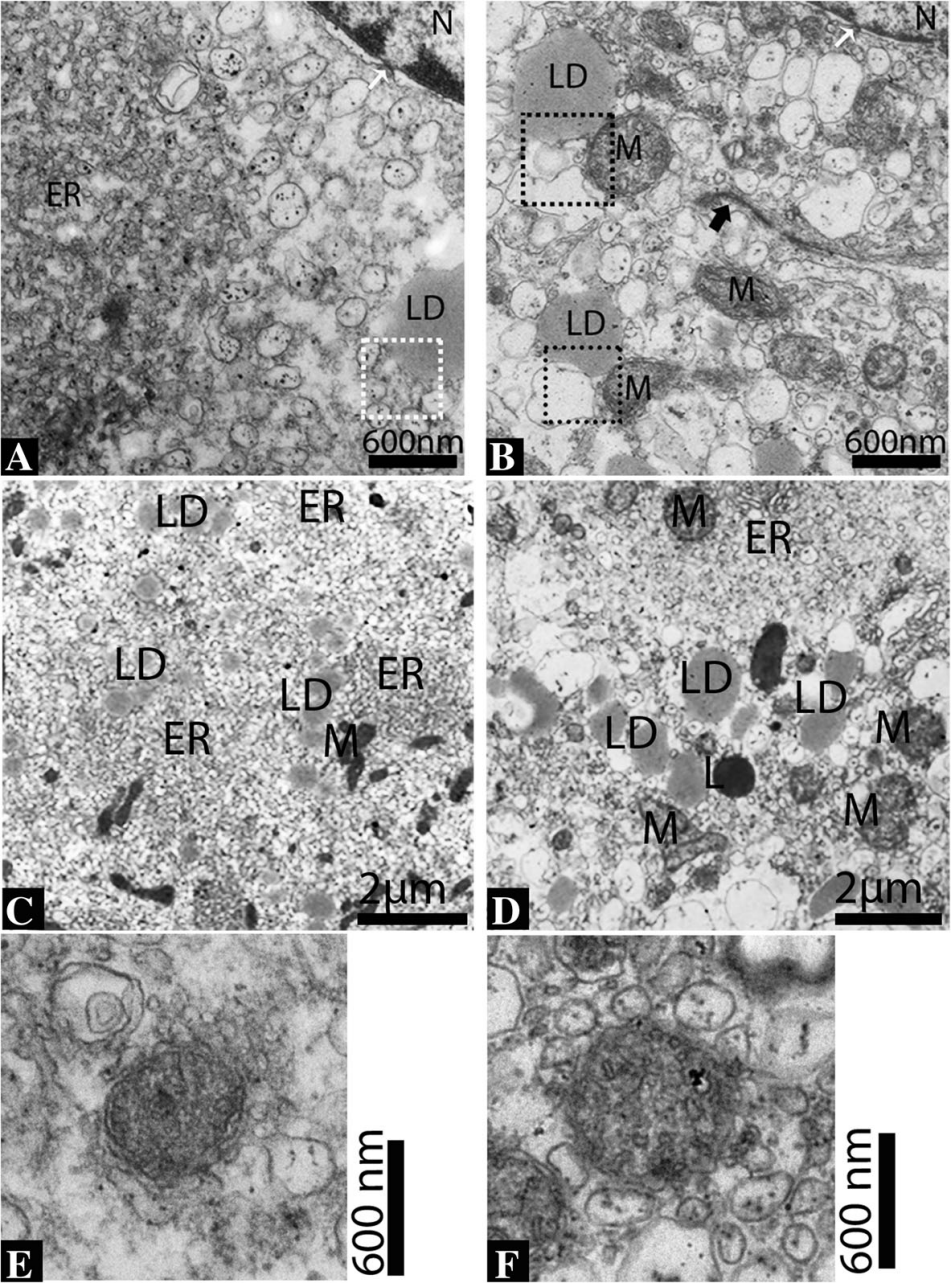
Ultrastructure of the Leydig cell. Abundant vesicular endoplasmic reticulum (**a**), and tubular endoplasmic reticulum and mitochondria (**b**) appear close to the nucleus and also show the nucleus pores (white arrow). Lipid droplets exhibit closely with vesicular endoplasmic reticulum during reproductive phase (**c**). In hibernation, the tubular endoplasmic reticulum, mitochondria and lysosome appeared to be in contact with the lipid droplets (**d**). The endoplasmic reticulum in contact with a lipid droplet (white dotted square) and with a lipid droplet and the mitochondria (black dotted square). Tight junctions between Leydig cell are also seen (black thick arrow). Mitochondria with well-developed inter cristae tubules (**e-f**). N: nucleus; ER: endoplasmic reticulum; LD: lipid droplets; M: mitochondria; L: Lysosome. Scale bar = 600 μm (**a-b**), 2 μm = (**c-d**) and 600 μm (**e-f**)

In the reproductive phase, the LC displayed round eccentric nuclei with prominent nucleoli. The nucleoli possessed fine, electron dense, granulated euchromatin and heterochromatin, with the latter adjacent to the nuclear envelope. The nuclear envelope displayed nuclear pores and was surrounded by the endoplasmic reticulum. The cytoplasm showed interdigitation spaces with finger-like cytoplasmic projections and tight junctions (Figs. 5a, c and 6a). At the perinuclear cytoplasm, the vesicular endoplasmic reticulum was abundantly dispersed and in contact with the mitochondria and lipid droplets (Fig. 6c and e). In hibernation, the nucleus of LC appeared elongated with an irregular membrane. The tubular endoplasmic reticulum, lipid droplets, mitochondria and lysosomes were observed as predominant organelles in the LC cytoplasm during this period. The mitochondria were observed in the dividing state and in contact with the endoplasmic reticulum. (Fig. 5b and d). At the perinuclear cytoplasm, the tubular endoplasmic reticulum, mitochondria and lipid droplets were found to be in-contact with another (Fig. 6b and d).

**Fig. 7:**
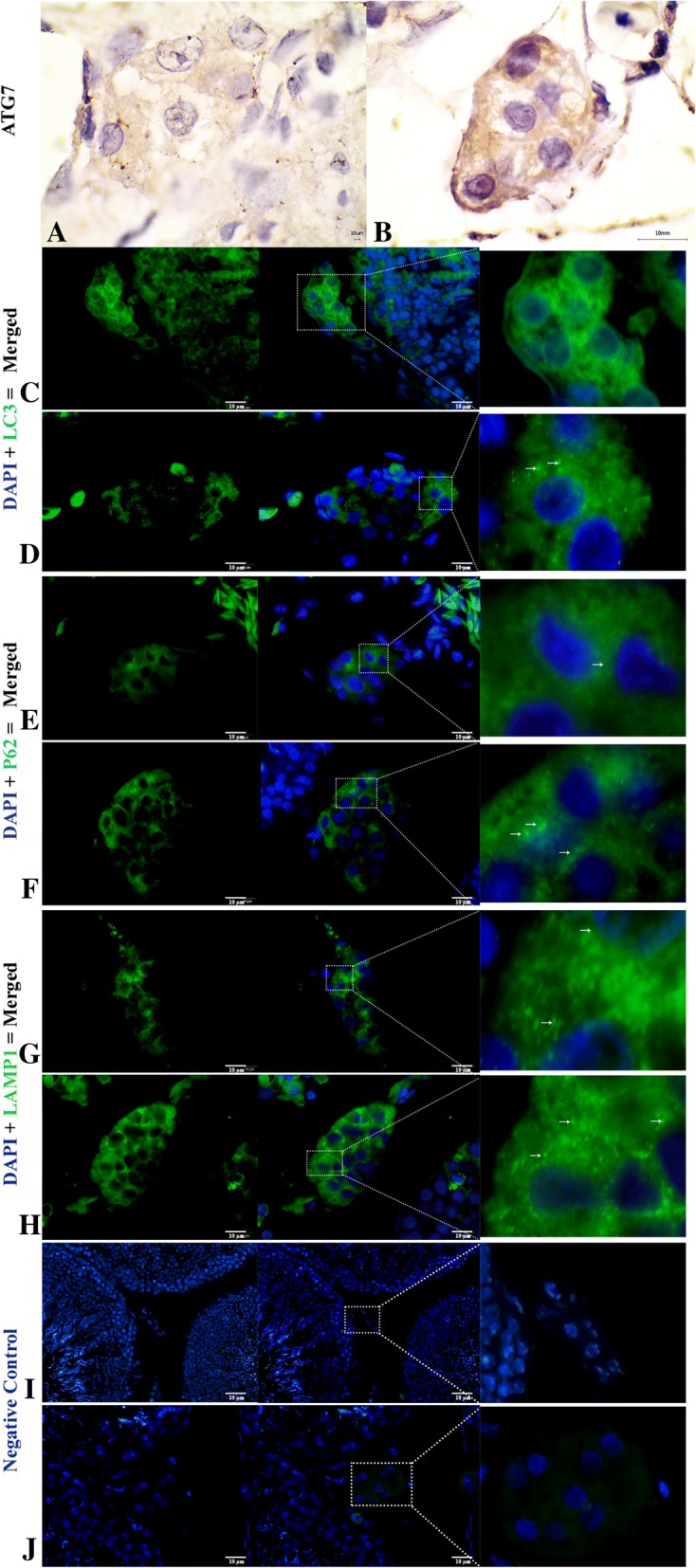
Immunofluorescent localization of autophagy markers in Leydig cells of the Chinese soft-shelled. Immunohistochemistry of ATG7 positive reactions in the Leydig cell (**a-b**). Immunofluorescence labeling of LC3, p62 and LAMP1 in the Leydig cell during reproductive (**c**, **e** and **g**) and during hibernation phase (**d**, **f** and **h**). Negative control; Reproductive (**i**) and hibernation phase (**j**). (White arrow): indicates positive localization. The higher magnification is illustrated by the rectangular area. Scale Bar = 10 μm (**a-h**)

**Fig. 8:**
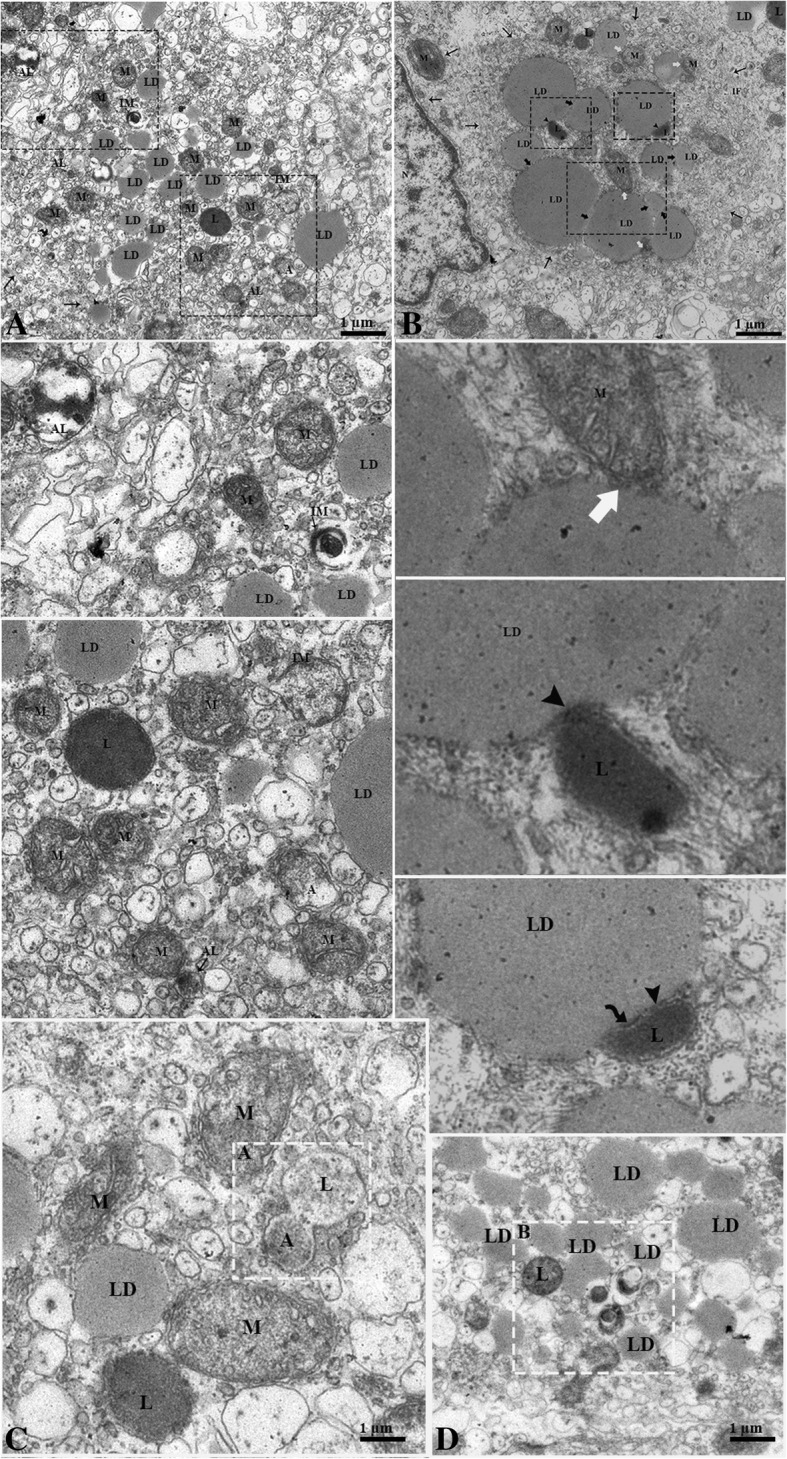
Electron micrograph of lipid droplet mobilization during hibernation. (**a**) The Leydig cell contains numerous small lipid droplets that appear closely to the lysosome and mitochondria. (**b**) Large lipid droplets in contact with each other (black thick arrow) and with the lysosome (black head arrow) and mitochondria (white thick arrow). A curved arrow indicates the autophagic tube, (black arrow) intermediate filaments, (black rectangular area) autolysosome, autophagosome and isolated membrane. The white rectangular area indicates the (**a**) fusion of autophagosome and lysosome, and the (**b**) lysosomal engulfing of the LD. The illustration is of a higher magnification. LD: Lipid droplets; M: Mitochondria; L: Lysosome; A: Autophagosome; AL: Autolysosome; IM: Isolated Membrane. Scale bar = 1 μm (**a-d**)

The autophagy markers evaluated by the immunohistochemistry of ATG7 signals were stronger during hibernation and weaker in reproductive phase. Moreover, LC3, p62 and LAMP1 had a strong spot-like immunosignals by immunofluorescence and were stronger during hibernation than the reproductive phase (Fig. 7). Additionally, the ultrastructure revealed that an extensive network of intermediate filaments surrounds the numerous large lipid droplets. These lipid droplets were in contact with each other as well as with the mitochondria and lysosomes. Lysosomes were detected in contact with the LD, showing an autophagic tube and the engulfing of the LD. Several small lipid droplets, mitochondria and lysosomes were observed around the phagophore, within the LC during development of the autophagosome and autolysosome. (Fig. 8a and b).

We further summarized the characteristic features of the Leydig cells during the annual reproductive cycle of the Chinese soft-shelled turtle in the Table 2.

**Table 2:** Ultrastructural Features of Leydig Cells during annual reproductive cycle of Chinese soft-shelled turtle

Features	Reproductive phase	Hibernation phase
Cluster Area / mm^2^	0.72 ± 0.1	1.5 ± 0.2
Nucleus	Round with smooth nuclear membrane (diameter = 7.1 ± 0.24 μm)	Oval to elliptical with irregular nuclear membrane (diameter = 6.9 ± 0.35 μm)
Lipid droplets	Few (diameter = 0.84 ± 0.027 μm)	Numerous (diameter = 1.1 ± 0.08 μm)
Mitochondria	Tubulo-vesicular cristae	
SER	Tubule-vesicular	Mainly tubular
Surface	Less Interdigitation space (finger like protrusions)	More Interdigitation space (finger like protrusions)
Arranged	Cluster	Cluster

## Discussion

The testicular morphology of the Chinese soft-shelled turtle was similar to that previously reported by our research group and in other reptiles [9, 28–30]. The dissociated reproductive pattern in the soft-shelled turtle indicates a seasonal activity in the tubular and interstitial compartments. The active reproductive phase was from May to Oct, and the quiescent or hibernation phase was from Nov to April [31]. During the quiescent phase, the seminiferous tubules regressed due to a single cohort releasing of spermatozoa into the epididymis. A parallel pattern was also found in *Trionyx sinensis*, which suggested the recrudescence of spermatogenesis [32]. Contrarily, mammals (rodents, monkeys, and human) produce sperm stored in the epididymis until ejaculation [33, 34]. After ejaculation, the principle activity of the Sertoli cell is removing the residual spermatids by apoptosis in mammals, while in reptiles, this occurs by entosis [29, 35, 36]. The interstitium cell’s fibroblasts and telocytes showed a communication via vesicles with the LC, suggesting cell to cell vesicles communication, hence these cells influenced the Leydig cell (LC) shape and testosterone production [37–39]. Hereafter, in past few decades the role of vesicles in cell-to-cell communication are of great interest in shaping their local environment by releasing factors that either effect adjacent cells or manipulate the biochemical properties of extracellular milieu [40]. The seasonal variation in the LC population is the indicative of interstitial precursor cells, that divide, then differentiate and replenish the LC numbers during testicular seasonal cycle [41]. Whereas the LC area related with steroidogenic activity based organelles such as SER that associated with androgen synthesis [42]. In contrast with present study results, In adults’ rats, the significant increased numbers and area of Leydig cell treated with hCG (human chorionic gonadotrophin) and LH (Luteinizing hormone) was reported [43]. While in the seasonal Golden hamster the LCs area was 30% reduced during inactive phase after photoinhibition [44] whereas in bat, the LCs became hypertrophied on the renewal of spermatogenesis [45]. During hibernation, the LC showed 3β-HSD activity and abundant tubular endoplasmic reticulum (ER) by IHC and TEM, which suggested an increased area in *Chrysemys picta* and *Chelydra serpentine* [30, 46]. These findings are also consistent with temperate-zone reptiles such as the lizard during different phases of the reproductive cycle [47]. The LC in turtles have the potential to become steroidogenic at any time of the year [48] and are known to contain tubular or SER at all times [46]. During reproductive activity, a similar consistency was found in present study by the weak expression of 3β-HSD and the existence of tubule-vesicular ER by TEM in the LC, which indicated a persistent steroidogenic activity, whereas in the LC of mammals (hamster, rat, rabbit, dog, guinea pig), the amount of SER was related to the testosterone secretion [49]. Moreover, we observed numerous lipid droplets (LD) within the LC by ORO staining during hibernation. The strong immunopositive reaction of vimentin (intermediate filament) and their appearance around the LD by TEM reflected the integrative role of intermediate filaments within the LC for steroid biosynthesis by facilitating the LD, and these LDs are a source of cholesterol for steroidogenesis [50]. In mammals, a similar constant of vimentin positivity was reported [51] and an in-vitro analyses presumed the facilitation of LD for the mitochondria [52]. As a result, the direct contact of the mitochondria with the LD facilitates its expansion in mammals [53], which was morphologically (TEM) observed in the current study.

Our study, the first for a non-mammalian vertebrate, illustrates the lipophagy within the Leydig cell of the Chinese soft-shelled turtle, which has previously been well studied in mammals by using the traditional methods of electron microscopy [26, 27] and autophagic markers (ATG7, LC3 and p62). We observed that, phagophore appeared close to the LDs during hibernation, which is the initial step in autophagosome formation [54] through the expansion of the endoplasmic reticulum [16]. The autophagosome then fused the with lysosome to develop the autolysosome [16]. In an In-vitro study of the LC, this phenomenon was associated with steroid biosynthesis by providing cholesterol to the LD and was known as lipophagy [14]. This phenomenon included lysosomal lipolysis to release fatty acids to meet functional demands [55, 56]. Hence, the current findings provided clear evidence of an autophagic to autolysosome (Fig. 8). Fascinatingly, we found lysosomes attached to LDs forming the autophagic tube and engulfing LDs (Fig. 7c and d, and 8). In yeast, micro-autophagy by autophagic tube engulfment has been described, but this has not yet been well characterized in other eukaryotes [17]. An in-vitro study using HeLa cells, concluded that LAMP1 and LAMP2 bind with cholesterol and are involved in cholesterol export [25]. Thus, for the TEM and immunofluorescence results, we suggest that macro-autophagy along with micro-autophagy were involved in the lipid metabolism. However, the lysosome is the endpoint of numerous vesicle trafficking pathways including for endocytosis, phagocytosis and autophagy [57]. To associate the function and activity of different autophagy types and abundant mitochondria, the lysosome and their attachment to LDs were observed during hibernation. This findings reflects that lipid metabolism releases the endogenous energy for steroidogenesis by different ways because the LDs are reserved as energy stock [58]. Furthermore, our findings demonstrate that the expression of specific autophagic markers are strongly expressed in the LC during hibernation compared to the reproductive phase. It has been suggested that the autophagy level was higher than other non-steroid-producing cells in mice and rats [27, 59] because of the increased cellular demand for autophagy imposed by the high turnover rate of steroid producing apparatus components [15]. Therefore, our autophagy marker and TEM findings are in line with the alteration of autophagy activity in turtle LC during the annual reproductive cycle.

## Conclusion

In conclusion, the Leydig cells in the Chinese soft-shelled turtle, *Pelodiscus sinensis* possessed a dynamic steroidogenic activity (active 3β-HSD), tubulo-vesicular ER, lipid droplets and tubular mitochondria during hibernation compared to the reproductive phase. Moreover, the current study provides novel morphological evidence on the involvement of lipophagy in mobilization of the LDs within the LC by two different modes (macro- and micro-phagy). This research indicates a new role for lipid metabolism in the LC of the turtle during the steroidogenesis process by autophagy.
